# Factors Associated with Preventable Emergency Department Visits for Nontraumatic Dental Conditions in the U.S.

**DOI:** 10.3390/ijerph16193671

**Published:** 2019-09-30

**Authors:** Pearl C. Kim, Wenlian Zhou, Shawn J. McCoy, Ian K. McDonough, Betty Burston, Marcia Ditmyer, Jay J. Shen

**Affiliations:** 1 Department of Health Care Administration and Policy, School of Public Health, University of Nevada, Las Vegas, NV 89154, USA; pearl.kim@unlv.edu (P.C.K.); betty.burston@unlv.edu (B.B.); 2 Department of Dental Medicine, School of Dental Medicine, University of Nevada, Las Vegas, NV 89106, USA; wenlian.zhou@unlv.edu (W.Z.); marcia.ditmyer@unlv.edu (M.D.); 3 Department of Economics, Lee Business School, University of Nevada, Las Vegas, NV 89154, USA; shawn.mccoy@unlv.edu (S.J.M.); ian.mcdonough@unlv.edu (I.K.M.)

**Keywords:** oral health, dental care, emergency departments, disparity

## Abstract

This study was designed to examine national trends and evaluate social determinants of health that were associated with the provision of dental services in emergency rooms in the United States between 2007 and 2014. A pooled cross-sectional database of emergency department (ED) visits combined the 2007–2014 waves of the Nationwide Emergency Department Sample. A total of 3,761,958 ED visits with dental conditions were extracted and the principal diagnosis was identified. A series of modified Poisson regression models were used to assess the relationship between patient sociodemographic factors and hospital characteristics, and the likelihood of visiting the ED for a nontraumatic dental reason. Unadjusted descriptive results indicated that there was no apparent increase in the percentage of patients who visited an ED with nontraumatic dental conditions (NTDCs) between 2007 and 2014. The greatest users of EDs for NTDCs were among those who were uninsured and Medicaid beneficiaries relative to persons privately insured. ED visitors were more likely to reside in lower socioeconomic areas (when compared with visitors in the top quartile of the income distribution). Patients in all other age groups were more likely to seek care in an ED for NTDCs relative to those 65 years of age or older. Multiple strategies are required to reduce the use of EDs for routine dental care. This approach will require an interprofessional dialogue and solutions that reduce barriers to receiving dental care.

## 1. Introduction

Emergency departments (ED) play an extremely crucial role in the United States health care system, especially for those who are underinsured or uninsured. Extreme overcrowding in EDs is now an issue of serious concern for it can result in reduced quality of care, delays in treatment, and increases in healthcare costs [1,2]. Much of this overcrowding can be attributed to the use of the ED for nonurgent or preventable health conditions which can be treated in a traditional medical office setting [3,4]. Generally, dental conditions are treated in dental offices. However, dental-related ED visits are on the rise [3,5,6]. Between 2000 and 2010, dental-related ED visits nearly doubled in the United States (U.S.). There were 1.1 million ED visits in 2000. This number had increased to 2.1 million in 2010 and has continued to increase [6]. On average, the costs associated with these visits range from one to two billion dollars annually in the U.S. [7]. Research has shown that approximately 1.5% of ED visits across the U.S. annually are dental care related issues. When data on these visits are analyzed, they reveal that the vast majority of those visits are for nontraumatic dental conditions (NTDCs) that can commonly be treated in a dental office [7]. Dental procedures are seldom performed in an ED. This results in palliative care being provided rather than definitive care [8]. As a result, 90% of dental-related ED visits result in prescription medication to manage pain and infection rather than appropriate dental procedures. This is because EDs are not equipped to provide extensive dental care [7]. Consequently, the majority of these visits require follow-up care with a dentist [9]. With the increase in the global burden of periodontal disease, oral cancer, and dental caries by an average of 45.6% from 1990 to 2010, it is anticipated that ED visits for NTDCs will rise. This trend parallels ED use for other major noncommunicable diseases such as diabetes [10].

Socioeconomically disadvantaged and medically underserved patients are especially vulnerable to resorting to ED visits for dental care [3,5]. Various barriers such as lack of insurance, living in a low-income area, and other demographic and socioeconomic factors have been shown to be associated with dental-related ED visits across the U.S. [8]. Dental care utilization among low-income children increased in 47 states between 2000 and 2010 [11]. However, for low-income adults, utilization declined and remained low in almost all states during that same time period. This trend is associated with significant reductions in adult dental benefits in Medicaid programs [11]. In 2012, the number of ED visits for dental conditions was estimated to be one person every 15 seconds at a cost to the U.S. healthcare system of approximately $1.6 billion annually. This represents an average cost of $749 per visit [11].

Although a rise in dental-related ED visits continues, gaps by demographic and socioeconomic factors that impact health have remained constant over time. Nonetheless, significant gaps in current literature regarding NTDCs in EDs remain. Currently, there are a limited number of studies that analyze nationally representative datasets in relation to dental-related ED visits. There have been few studies that have analyzed actual hospital data or cross-referenced specific patient data. Rather, past studies have relied more on survey data. Such datasets are characterized by potential recall and reporting bias. The use of survey data is inclusive of other shortcomings that prevent accurate depictions of the current burden [12,13,14,15]. The purpose of this study is to examine national trends and evaluate social determinants of health that are associated with the increased burden on EDs of providing dental services in the U.S. between 2007 and 2014. Findings from the study may assist in developing strategies and programs to alleviate ED crowding and reduce the health disparities for dental care in lower income populations and communities.

## 2. Materials and Methods 

We constructed a pooled cross-sectional database of ED visits by combining the 2007–2014 waves of the Nationwide Emergency Department Sample (NEDS). A total of 3,761,958 ED visits with dental conditions (traumatic or nontraumatic) as the principal diagnosis were extracted as the sample for the analysis. We created a dichotomous outcome variable equal to 1 for a NTDC and 0 otherwise. Of the total visits, 3,423,382 were identified as NTDCs (91.0%). Table 1 presents descriptive statistics for the data used in the analysis. 

We estimated a series of modified Poisson regression models to directly obtain estimates for relative risk in order to assess the relationship between patient sociodemographic factors and hospital characteristics (independent variables), and the likelihood of visiting the ED for a nontraumatic dental reason. In particular, we were interested in how the likelihood of ED utilization for a NTDC changed given an incremental change in selected covariates as reflected by relative risk measures obtained from the modified Poisson framework. 

The set of observable patient characteristics included categorical variables for 7 age groups (< 2 years old, 2–11, 12–19, 20–34, 35–49, 50–64, and >65). Sex, insurance status (Medicare, Medicaid, private insurance, and uninsured), and level of median income by zip code and quartiles were also included. Hospital characteristics included urban/rural status, ownership (public, not-for-profit, for-profit), teaching hospital status, and trauma designation. The study controlled for underlying trends in the data by including a linear time trend. In the first stage of our analysis, we estimated a multivariate logistic regression model by pooling the data over all time periods and controlling for all variables listed above.

For each sociodemographic factor, the project assessed disparities in the likelihood of ED utilization for a NTDC among individuals within each class (e.g., higher vs. lower income quartiles, health insurance status, gender, and age group) and evaluated the persistence in any identified disparities over time. Coefficient estimates obtained from our baseline logistic model were used for assessing socioeconomic disparities in ED utilization for a NTDC. In the second stage of our analysis, we obtained sequential estimates of our baseline modified Poisson model by estimating the model separately for each sampled year. This approach allowed us to effectively investigate trends in disparities by reporting estimated relative risks for each socioeconomic variable of interest over time. Lastly, appropriate sample weights provided in the data were used throughout the analysis. 

## 3. Results

Unadjusted descriptive results (Table 1) indicated only minor variations in the percentage of patients who visited EDs with NTDCs between 2007 and 2014. Relative to age, the majority of these visits were young adults (20–34 years of age) and middle aged adults (35–49 years of age). Additionally, there was only minor observable changes in ED use patterns between males vs. females from 2007 to 2014, although slightly more females (~53%) sought help in ED for NTDCs than males (~47%). The greatest users of EDs for NTDCs were among those who were uninsured and who were Medicaid beneficiaries. There was a decrease to 32.9% in 2014 from 40.6% in 2013 among the persons who were uninsured and a large increase in EDs use by Medicaid beneficiaries. In 2014, the use rate was 40.6% relative to a rate of 34.5% in 2013. Relative to income, the greatest overall use of EDs for NTDCs in 2014 was by individuals who resided in the socioeconomic areas that housed persons in the lowest 25% of the population.

Key findings from the modified Poisson regression models are presented in Table 2. With the exception of persons ages 65 and older (the reference group), all age groups were more likely to seek care in an ED for NTDCs, with the greatest likelihood being in children less than 2 years of age. This group was 38.7% more likely to use EDs for NTDCs than was the case for persons age 65 and older. While the differences in gender were statistically significant, the clinical significance was very small (Females RR = 0.98; Males RR = 1.00, *p* < 0.001). 

When compared to those with private dental insurance (the reference group), those persons with Medicaid and no insurance were more likely to seek care in the ED for NDTCs (6.0% and 6.5% respectively). 

With respect to median income, persons in the bottom 75% of the income distribution were more likely than those residing in the top quartile of the income distribution (the reference group) to seek care in the ED for NTDCs. However, and similar to gender, these differences were only slightly less with the 1^st^ quartile at 1.041 (*p* < 0.001), 2^nd^ quartile at 1.039 (*p* < 0.001), and the 3^rd^ quartile at 1.029 (*p* < 0.001) (Table 2). 

The data were also analyzed to determine whether differences in the use of EDs for NTDCs existed based on characteristics of the hospital as assessed by hospital region, hospital ownership, rural vs. nonrural status, and teaching vs. nonteaching hospital types. With respect to geographical differences in hospital region, the differences in relative risk were very slight, with those in the Southern region of the U.S. being ~1.7% (*p* < 0.000) more likely to utilize the ED for dental services than was true for hospitals in the West (the reference group). This relationship was also observed when comparing differences in rural (RR = 0.99, *p* < 0.023) vs. metropolitan areas (the reference group). Hospitals that were public, teaching facilities (RR = 0.99, *p* < 0.718), or nonprofit, nonteaching hospital (reference group) had results that were statistically insignificant relative to their counterparts.

## 4. Discussion

The upward trend in the use of EDs for NTDCs was observed across the U.S. and Canada where ED visits have been increasing for the past several years [14,15,16,17]. This increase created tremendous challenges for efficient healthcare delivery national wide [17,18]. Our study indicates that while there is an upward trend in the total number of dental-related ED visits, the percentage of NDTCs remains flat between 2007 and 2014. Furthermore, differences among demographic and socioeconomic factors remained unchanged between 2007 and 2014 even after considering the increase in Medicaid enrollment since 2012. 

### 4.1. Demographic Factors: Age and Gender 

Our study revealed that ED visits for dental conditions were greatest among children less than 2 years of age, among young adults (ages 20–34) and middle aged adults (ages 35–49), followed by individuals less than 20 years old and individuals in the age range 50–64 when compared with individuals 65 years or older. Thomas Wall studied the national trends for ED NTDC visits from 1997/1998 to 2007/2008. He found that the largest increase in the number of dental ED visits per 1000 persons was in the young adults group of 20–34 years old, followed by the group in the age range 35–49 [3]. This is similar to the findings from this study and other findings [8]. A possible reason that young adults are more likely to go to the ED for NTDCs could be that dental care is not a federally mandated benefit for adult Medicaid beneficiaries. A number of states have reduced or eliminated Medicaid dental benefits for adults due to budget issues. Young adults and others who lack access to the private practice dental delivery system may be forced to use physician’s offices, hospital EDs, or other ambulatory care settings for their dental needs [3]. One major driver of demand is population demographic changes. In 2015, there were 30 million visits for children aged 18 years or younger, with a vast majority being treated and released [19]. The reasons could be due to many factors including limited access to primary care; an absence of confidence in primary care; parents perceived urgency; convenience; views of family, friends, or other health professionals; and the parental belief that their child’s condition requires the resources and facilities offered by a particular healthcare provider [20]. The data also suggest that females are slightly less likely to visit the ED for NTDCs compared to their male counterparts. 

### 4.2. Insurance Status

Insurance status is one of the primary factors that was investigated regarding NTDCs and ED visits. Results from our study suggest that inadequate access to dental care is a main predictor for NTDC-related visits to the ED. Based on the study results, Medicaid and “no insurance” individuals are both associated with higher odds of visiting the ED for NTDCs relative to those with private insurance. It should be noted that there are no provisions for dental insurance in Medicare for persons over the age of 65. Therefore, these individuals can be considered as being “without insurance” unless they have purchased additional dental insurance. These results supported previous studies’ findings [4,8].

Lack of insurance or fluctuations in insurance status are associated with increased use of emergency services and reduced use of nonhospital health care services [8,21]. ED visitors with no insurance and Medicaid were more likely to be associated with a nontraumatic dental problem compared with those who were commercially insured. The elimination of state adult Medicaid dental benefits may have also led to increases in ED dental visits during the period of the study. Thus, findings from this study underscore the importance of comprehensive dental coverage for Medicaid beneficiaries [8]. Uninsured individuals, due to either lack of employer-sponsored insurance, the financial inability to afford a private dental insurance program, and/or an income level that disqualifies them for government insurance programs, are more likely to not access preventive dental care [22]. Accordingly, when they have emergent dental care needs, they are more likely to seek care in an ED.

### 4.3. Income

Previous studies revealed that low-income, socioeconomically vulnerable individuals are at greatest risk for ED dental visits [8,21,22]. Our study results showed that the individuals in the lower 75% of the income distribution are more likely to go to the ED for their NTDCs when compared to those individuals in the top 25% of income earners. The possible explanation could be that for low-income adults, in the absence of dental benefits, cost is the main barrier to accessing regular dental care. Therefore, the ED is seen as an alternative to address emerging dental pain and infection. Health policy may need revision to expand dental care access nationwide via both Medicaid and Medicare. 

### 4.4. Regional or Geographical Disparity

Our study found that people living in rural areas had a higher risk of visiting the ED for a NTDC relative to their urban counterparts. This could, of course, be due to a lack of dentists serving the rural areas. It has been reported that residents of nonmetropolitan areas with unstable or no insurance coverage may be at particular risk for reduced access and use of selected health services relative to those living in metropolitan areas [22,23]. Additionally, certain regional differences were noted in our study. Individuals who lived in the Northeast, Midwest, and South were more likely to visit the ED for NTDCs than people living in the Western U.S. 

Stevens et al. [24] explored physical, economic, and psychological factors as well as standard demographic factors in characterizing the health care access problems among older patients (65+) presenting at hospitals in the Southeastern U.S. This observation may also be attributed to variations in access to primary dental services among different regions [4,24]. 

Additionally, our study found that the trend of ED visits with NTDCs remained unchanged between 2007 and 2014. This result is different from other findings. Lee et al. [6] found an increasing trend in ED dental visits over the years 2001 to 2008 in data from the National Hospital Ambulatory Medical Care Survey (NHAMCS). Pajewski and Okunseri [25], in their analysis of Wisconsin 2001–2009 Medicaid data that focused on follow-up treatment after NTDC ED visits among adult Medicaid patients, found a 43% increase in NTDC visits to EDs over the nine years of data. Although this difference may be interesting, there are several explanations about our different findings. First, both prior studies cited here used earlier data periods, 2001–2008 and 2001–2009, respectively, whereas our data period is more recent. Second, Lee and colleagues used the NHAMCS that is based on self-reported information, while the NEDS we used are more accurate in documenting ED visits. Third, Pajewski and Okunseri only studied the Medicaid patients in one state. More importantly, the upward trend they reported was largely due to the low ED use during 2001–2005 and a marked increase in ED visits from 2005 to 2006. In fact, looking at their 2007–2009 data that are overlapped with our data, one can see the ED visits with NTDCs showed a flat trend that is consistent with our findings. We believe that our findings of the unchanged trend reflect a more recent national trend, whose underlying factors merit further investigation. 

### 4.5. Public Health and Policy Implications

NTDCs can be addressed through regular dental care in a general dental setting. In the U.S., the current model of dental practice is general dentistry combined with the specialized disciplines that comprise comprehensive dental care. This model emphasizes primary and preventive care in order to promote efficient, cost-effective treatment while maintaining the continuity of care. 

ED care that only addresses symptoms without definitive care to alleviate the cause of oral problems results in patients often returning to EDs multiple times for the same problem [26]. ED visits, especially when repeated for the same problem, generate high costs to the patient, the public, insurance companies, and taxpayers, depending on the patient’s ability to pay [27]. The use of EDs for NTDCs may delay care for those patients who truly need emergency care. Besides increasing the dental workforce nationwide or correcting the maldistribution of the existing dentists to areas that are considered shortage areas, other potential solutions to this crisis include improving the dental benefits coverage under Medicaid. Additionally, the inclusion of dental coverage in Medicare may also contain and/or reduce this problem. Recruiting and retaining more dental providers who accept Medicaid is another possible solution. It is possible to develop some programs or facilities that can improve the efficiency and quality of care for patients with dental emergencies while reducing the burden on ED nationwide. One solution may be to establish and support community level, low-cost dental facilities or programs [28] to improve the populations’ access to dental care. Incorporating a dental emergency unit in a traditional ED setting is also a viable option [29]. Clearly, multiple strategies are required to reduce the use of ED for routine dental care. 

The study had several limitations. One potential limitation was that the assessment of the extent that disparities persist (or not) over time was not possible given the nature of our cross-sectional dataset. Another limitation was that information about race/ethnicity was not available in the NEDS (although race/ethnicity is often correlated with health insurance and other socioeconomic factors). The third limitation was being unable to link the data to more comprehensive information on patients’ socioeconomic environments (e.g., the Census data) that may affect disparities as well.

## 5. Conclusions

Although our study demonstrates no evident increase in national trends of ED visits for NTDC between 2007 and 2014, the study confirms that social determinants of health are associated with a high risk of using EDs for NTDCs. We found that Medicaid, uninsured patients, and low-income individuals were at greatest risk of more frequent NTDC-related ED visits. Although we found individuals living in rural areas were at a higher risk of using EDs for a NTDC than were their urban counterparts, further studies that explore regional factors that align with NTDCs-associated ED visits are needed. At minimum, this study was designed to facilitate interprofessional dialogue on solutions that reduce barriers to receiving regular dental care.

## Figures and Tables

**Table 1:** Characteristics of emergency department visits for nontraumatic dental conditions in the United States, 2007–2014.

Independent Variables	2007	2010	2013	2014	All 8 Years
N	426,514	468,213	473,044	505,263	3,761,958
NTDCs (%)	90.5	91.3	90.9	91.4	91.0
*Patient Characteristics*					
Age Group (%)					
Age less than 2	2.0	1.7	1.7	1.7	1.8
Age 2 to 11	5.0	4.3	4.7	4.8	4.7
Age 12 to 19	7.1	6.0	4.9	4.7	5.9
Age 20 to 34	50.1	51.7	50.3	49.7	50.6
Age 35 to 49	25.4	24.6	24.6	24.7	24.7
Age 50 to 64	7.7	8.9	10.5	11.0	9.3
Age 65+	2.8	2.8	3.3	3.5	3.0
Female (%)	53.3	52.9	53.0	52.8	52.9
Health Insurance Status (%)					
Medicare	7.1	7.4	8.6	9.0	8.0
Medicaid	27.8	32.3	34.5	40.6	33.3
Private Insurance	24.0	18.3	16.2	17.5	18.8
No Insurance	41.2	41.9	40.6	32.9	39.9
Median Income by Zip Code					
1^st^ Income Quartile	38.7	40.0	41.0	41.3	39.6
2^nd^ Income Quartile	29.5	31.2	29.9	30.7	30.6
3^rd^ Income Quartile	20.9	18.9	19.4	18.1	19.7
4^th^ Income Quartile	10.8	10.0	9.7	9.9	10.1
*Hospital Characteristics* (%)					
Rural Area	9.3	9.4	7.7	6.7	8.3
Region					
Northeast	20.6	18.8	17.9	17.1	18.7
Midwest	27.5	26.4	25.7	26.1	26.5
West	11.4	12.3	12.6	13.5	12.4
South	40.5	42.5	43.7	43.3	42.4
Ownership					
Public	8.2	6.8	7.1	7.3	7.5
Nonprofit	16.0	14.8	15.4	12.8	15.3
For-Profit	8.0	8.2	9.3	7.6	8.4
Teaching Hospital	33.9	37.3	40.7	50.6	38.6

Notes. Appropriate sample weights provided by the Healthcare Cost and Utilization Project (HCUP) were used in calculating the above descriptive statistics. Data are expressed as a percent unless otherwise noted. Percentages may not add to 100 because of rounding; N = number of national observations, weighted; NTDCs = Nontraumatic Dental Conditions.

**Table 2:** Determinants of nontraumatic dental condition diagnosis (N = 3,761,958).

Independent Variables	Relative Risk	95% CI	*p*-Value
Year Trend	0.997	[0.992–1.003]	0.325
*Patient Characteristics*			
Age group			
less than 2	1.387	[1.370–1.404]	0.000
2–11	1.286	[1.271–1.301]	0.000
12–19	1.260	[1.245–1.275]	0.000
20–34	1.341	[1.326–1.356]	0.000
35–49	1.325	[1.310–1.340]	0.000
50–64	1.260	[1.248–1.272]	0.000
65 and older (reference)	1.000		
Sex			
Female	0.978	[0.977–0.979]	0.000
Male (reference)	1.000		
Health insurance status			
Medicare	1.032	[1.027–1.036]	0.000
Medicaid	1.060	[1.056–1.064]	0.000
No Insurance	1.065	[1.062–1.069]	0.000
Private Insurance (reference)	1.000		
Median income by zip code			
1st Quartile	1.041	[1.034–1.048]	0.000
2nd Quartile	1.039	[1.033–1.046]	0.000
3rd Quartile	1.029	[1.023–1.035]	0.000
4th Quartile (reference)	1.000		
*Hospital Characteristics*			
Hospital region			
Northeast	1.016	[1.007–1.025]	0.001
Midwest	1.008	[1.000–1.016]	0.041
South	1.017	[1.010–1.023]	0.000
West (reference)	1.000		
Hospital ownership			
Public	0.986	[0.976–0.995]	0.004
For-Profit	0.997	[0.989–1.006]	0.526
Nonprofit (reference)	1.000		
Rural hospital			
Yes	0.990	[0.981–0.999]	0.023
No (reference)	1.000		
Teaching Hospital			
Yes	0.999	[0.993–1.005]	0.718
No (reference)	1.000		

Notes. CI = confidence interval; N = number of observations; in all cases sample weights provided by HCUP were used in the estimation.
